# Supplementary material for: Meningeal lymphatic dysfunction exacerbates traumatic brain injury pathogenesis
Source: Nat Commun. 2020 Sep 10;11:4524. doi: 10.1038/s41467-020-18113-4 (PMC7483525; doi:10.1038/s41467-020-18113-4)
Supplement: Supplementary file 1 — Supplemenatry Information [file 41467_2020_18113_MOESM1_ESM.pdf]

Supplementary Information

Meningeal lymphatic dysfunction exacerbates traumatic brain injury pathogenesis

Bolte et al.

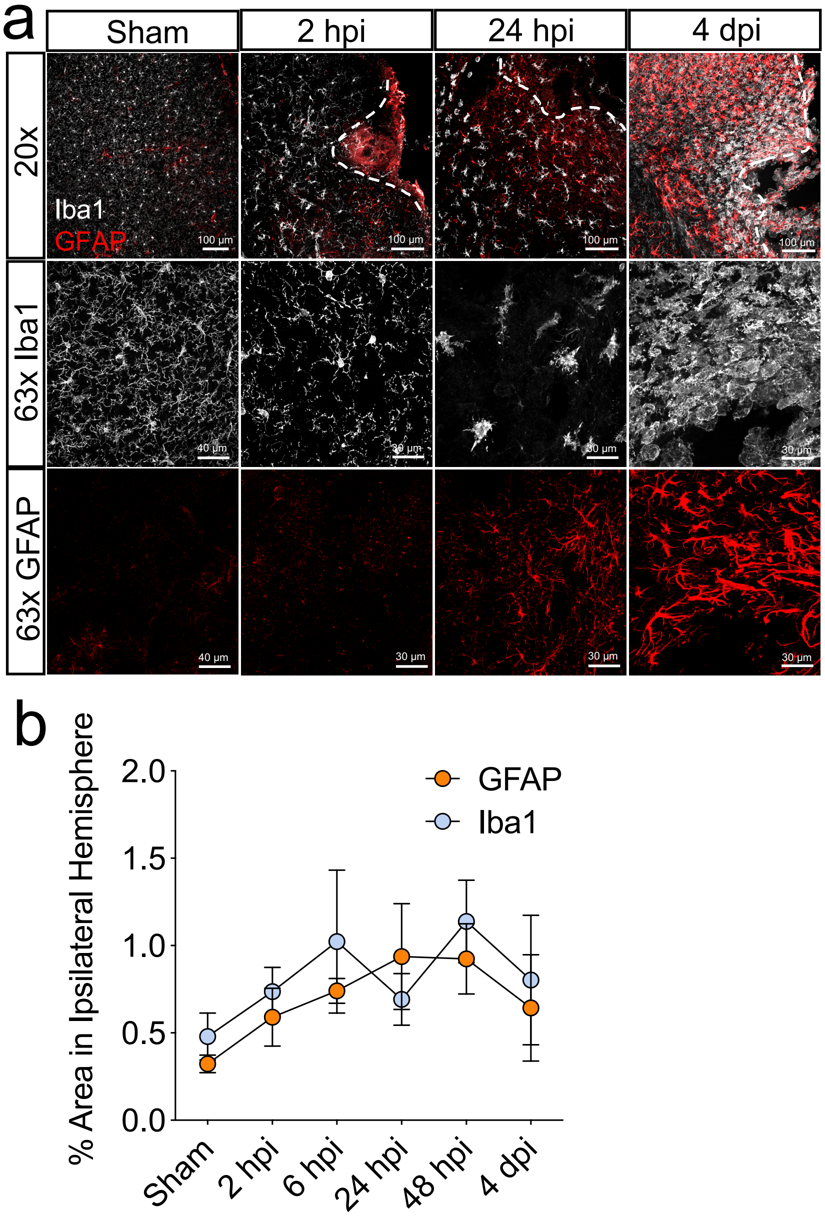

Supplementary Figure 1. Mild closed-skull TBI results in modest increases in gliosis. Brains were harvested at various time points after TBI and stained for GFAP and Iba1. a) Representative images of immunofluorescence staining and b) percent area coverage of Iba1 and GFAP in the brain hemisphere containing the TBI lesion site (Sham n=4, 2 hours n=4, 6 hours n=2, 24 hours n=6, 48 hours n=2, 4 days n=2; pooled data from 2 independent experiments). Dashed lines indicate edge of lesion. All n values refer to the number of mice used and the error bars depict mean ± s.e.m. dpi, days post injury; hpi, hours post injury. Source data (b) are provided as a Source data file.

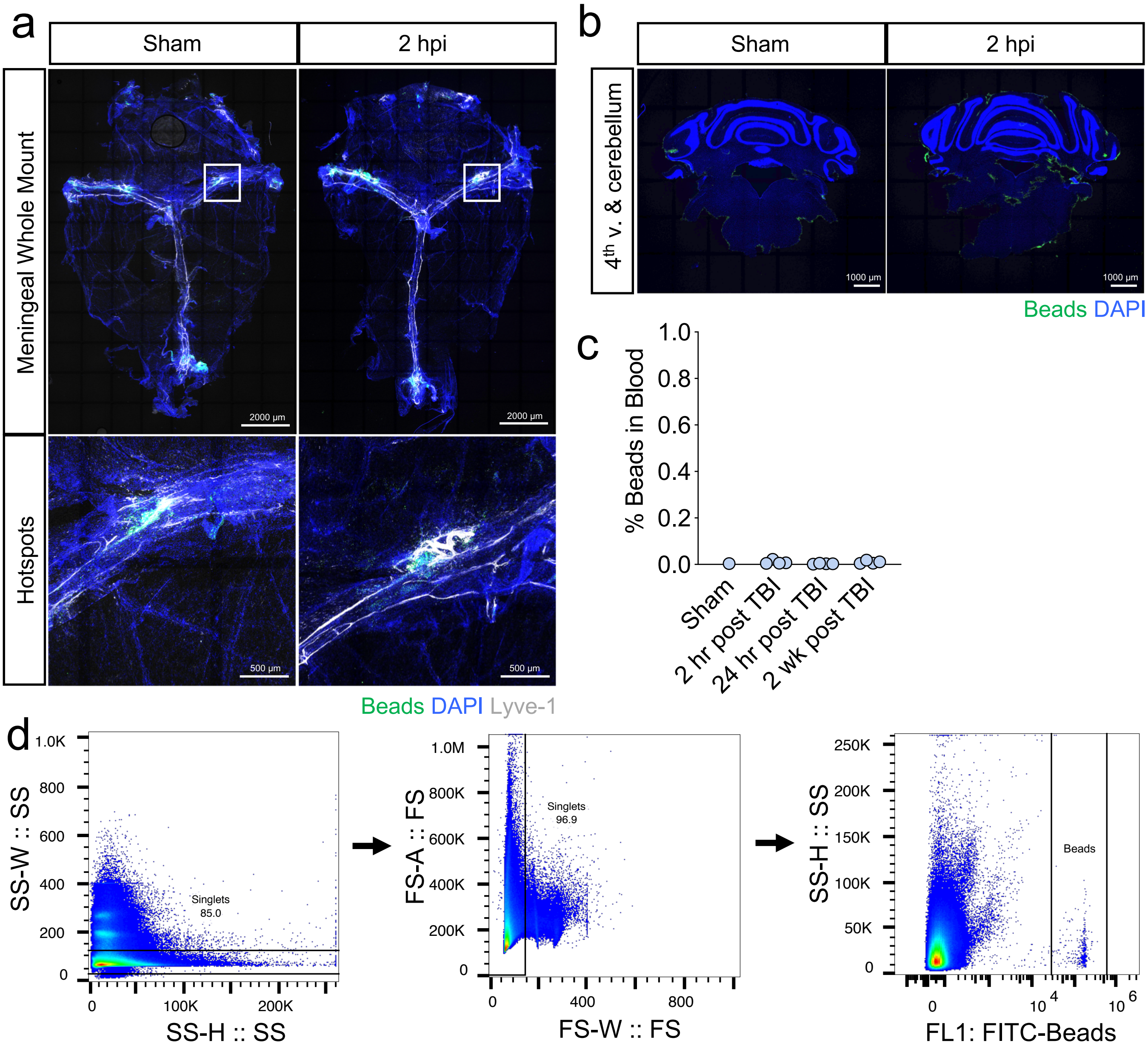

Supplementary Figure 2. Bead localization following i.c.m. injection. Mice received TBI or sham treatment and then received 0.5 µm fluorescent beads (green) by i.c.m. injection 2 hours later. Meningeal whole mounts and brains were harvested 2 hours after injection. a) Representative images of meningeal whole mounts stained with DAPI (blue) and Lyve-1 660 (grey) after TBI or a sham procedure and fluorescent bead injection. Solid box shows inset of bead uptake at the hotspot along the transverse sinus. b) Representative images of fluorescent beads in the fourth ventricle (4th v.) and cerebellum. Images (a,b) are representative of two independent experiments with similar results. c) Flow cytometry data depicting frequency of beads in the blood 2 hours, 24 hours, or 2 weeks post-TBI (Sham n=1, 2 hours n=4, 4 hours n=4, 2 weeks n=4; representative data from 2 independent experiments). d) Gating strategy for flow cytometry data to analyze the frequency of microbeads from total singlet events in blood. All n values refer to the number of mice used and the error bars depict mean ± s.e.m. hpi, hours post injury. Source data (c) are provided as a Source data file.

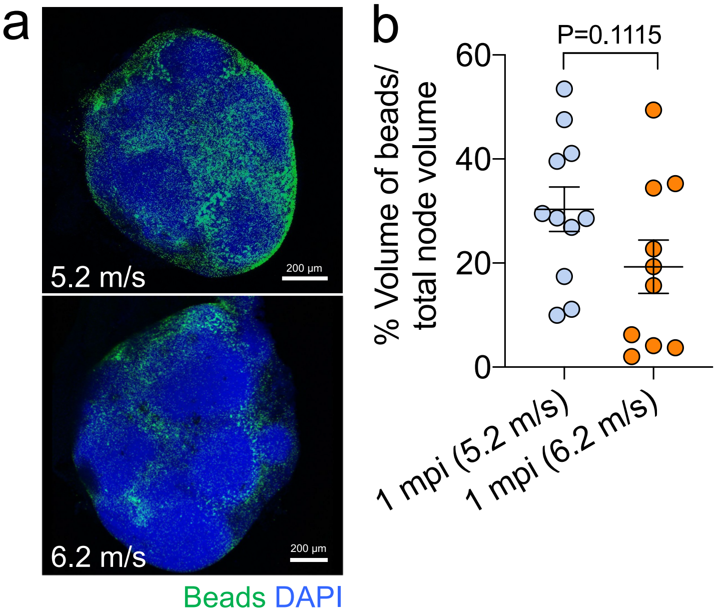

Supplementary Figure 3. Higher velocity TBI negatively affects bead drainage. Mice received TBI with an impact velocity of either 5.2 m/s or 6.2 m/s, and then one month later were injected i.c.m. with 0.5 μm fluorescent beads. dCLN were harvested 2 hours after bead injection and cleared according to the CUBIC protocol. a) Representative images of cleared dCLN and b) quantification of the percent volume of beads in the total node volume one month after injury (5.2 m/s n=11, 6.2 m/s n=10; pooled data from 2 independent experiments). All n values refer to the number of mice used and the error bars depict mean ± s.e.m. *P* value was calculated by two-tailed unpaired Student's t-test. mpi, month(s) post injury. Source data (b) are provided as a Source data file.

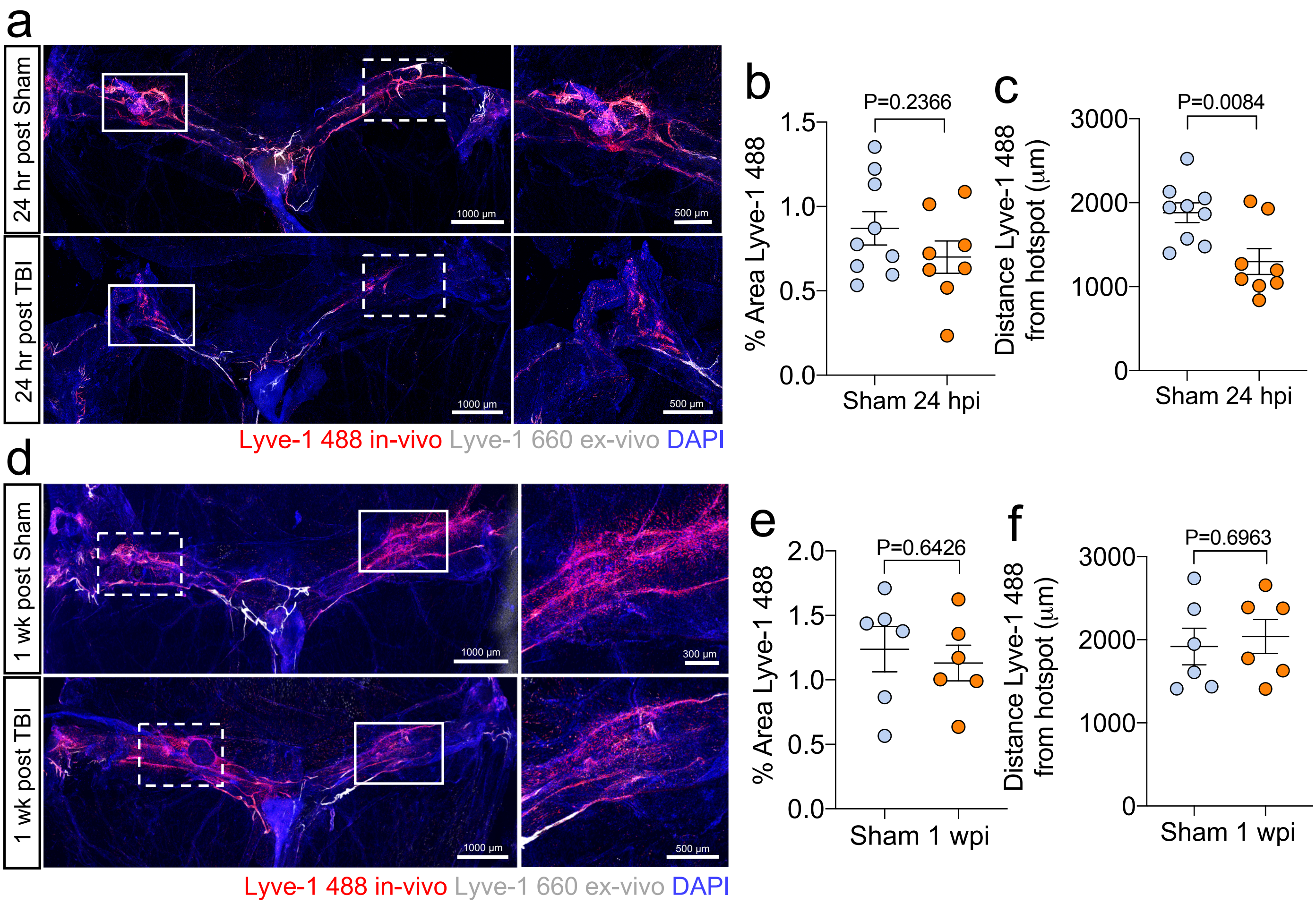

Supplementary Figure 4. Meningeal lymphatic uptake of CSF at hotspots is impaired after TBI. Mice received TBI or sham treatment and then fluorescently labeled anti-Lyve-1 488 antibodies (red) were injected i.c.m. into the CSF 24 hours or 1 week later. 15 minutes after injection of Lyve-1 488 antibodies (red), meningeal whole mounts were harvested and ex vivo stained with anti-Lyve-1 660 (grey). a,d) Representative images of meningeal whole mounts (a) 24 hours and (d) 1 week after TBI, stained for Lyve-1 488 (in vivo, red), Lyve-1 660 (ex vivo, grey) and DAPI (blue). Solid boxes show zoomed insets of the hotspots along the transverse sinus on the right. Dashed boxes indicate the other hotspots not featured in the insets. b,e) Percent area of Lyve-1 488 coverage at (b) 24 hours and (e) 1 week post-TBI. c,f) Distance traveled of Lyve-1 488 (in vivo, red) staining along the transverse sinus 15 minutes after injection (c) 24 hours and (f) 1 week after injury. a-c) (Sham n=9, 24 hours n=8; pooled data from two independent experiments) (d-f) (Sham n=6, 1 week n=6; data from one experiment). All n values refer to the number of mice used and the error bars depict mean ± s.e.m. *P* values were calculated by two-tailed unpaired Student's t-test. hpi, hour(s) post injury; wpi, week(s) post injury. Source data (b,c,e,f) are provided as a Source data file.

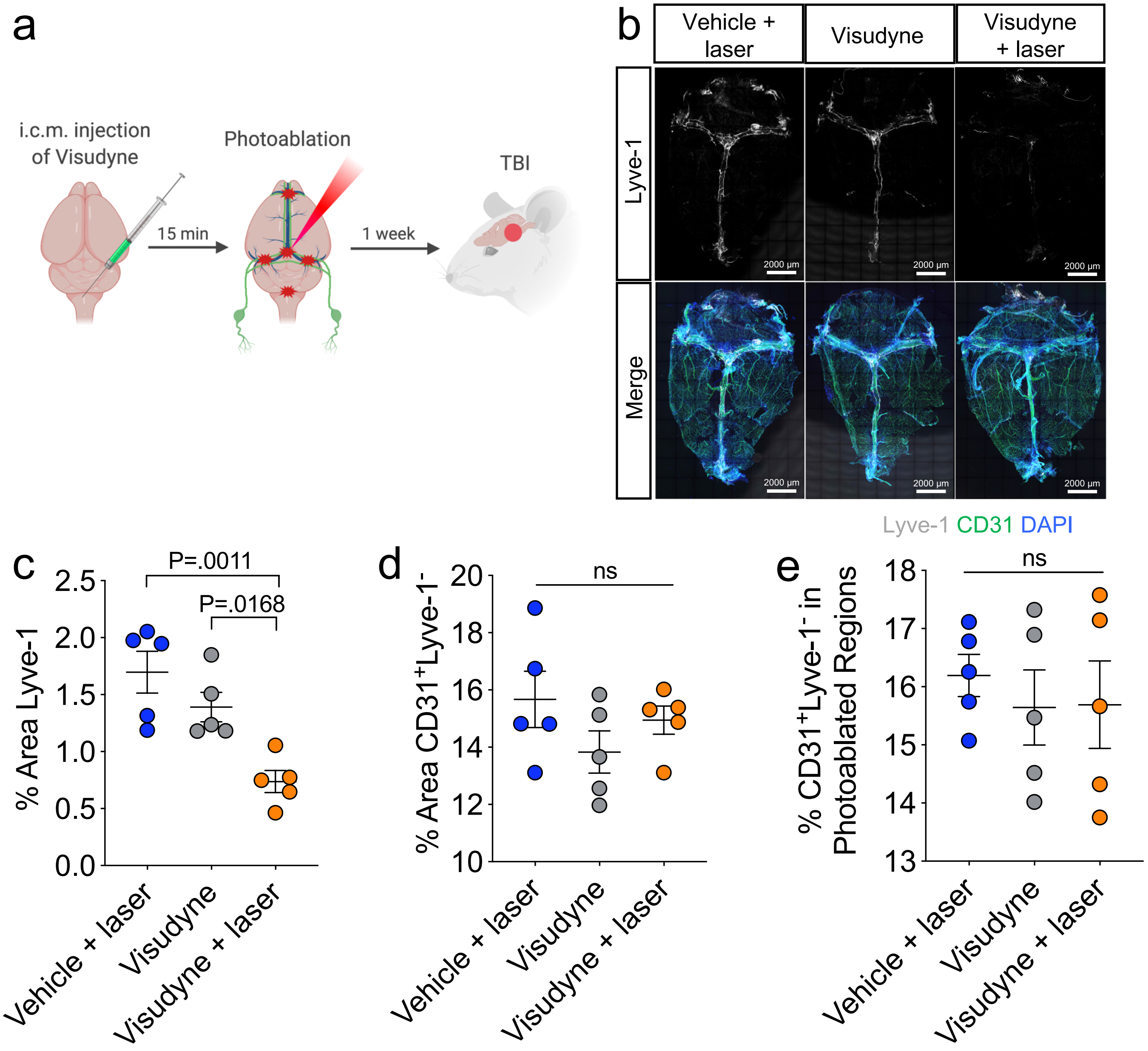

Supplementary Figure 5. Visudyne photoablation of the meningeal lymphatic vasculature. a) Schematic of experimental layout. Mice were subjected to an injection of Visudyne or vehicle i.c.m., and 15 minutes later a red laser was directed at 5 spots along the sinuses through the skull. b-e) Meningeal whole mounts were harvested 1 week post-photoablation and then were stained with Lyve-1 660 (grey), CD31 (green), and DAPI (blue) to assess lymphatic (Lyve-1^{+}) and blood (CD31^{+}Lyve-1^{-}) vasculature. Percent area coverage of (c) Lyve-1 660 or (d) CD31^{+}Lyve-1^{-} in meningeal whole mounts. e) Percent area coverage of CD31^{+}Lyve-1^{-} in regions specifically targeted by photoablation (n=5 for all groups; representative data from 3 independent experiments). All n values refer to the number of mice used and the error bars depict mean ± s.e.m. *P* values calculated by one-way ANOVA with Tukey's post hoc test (c,d,e). Source data (c,d,e) are provided as a Source data file.

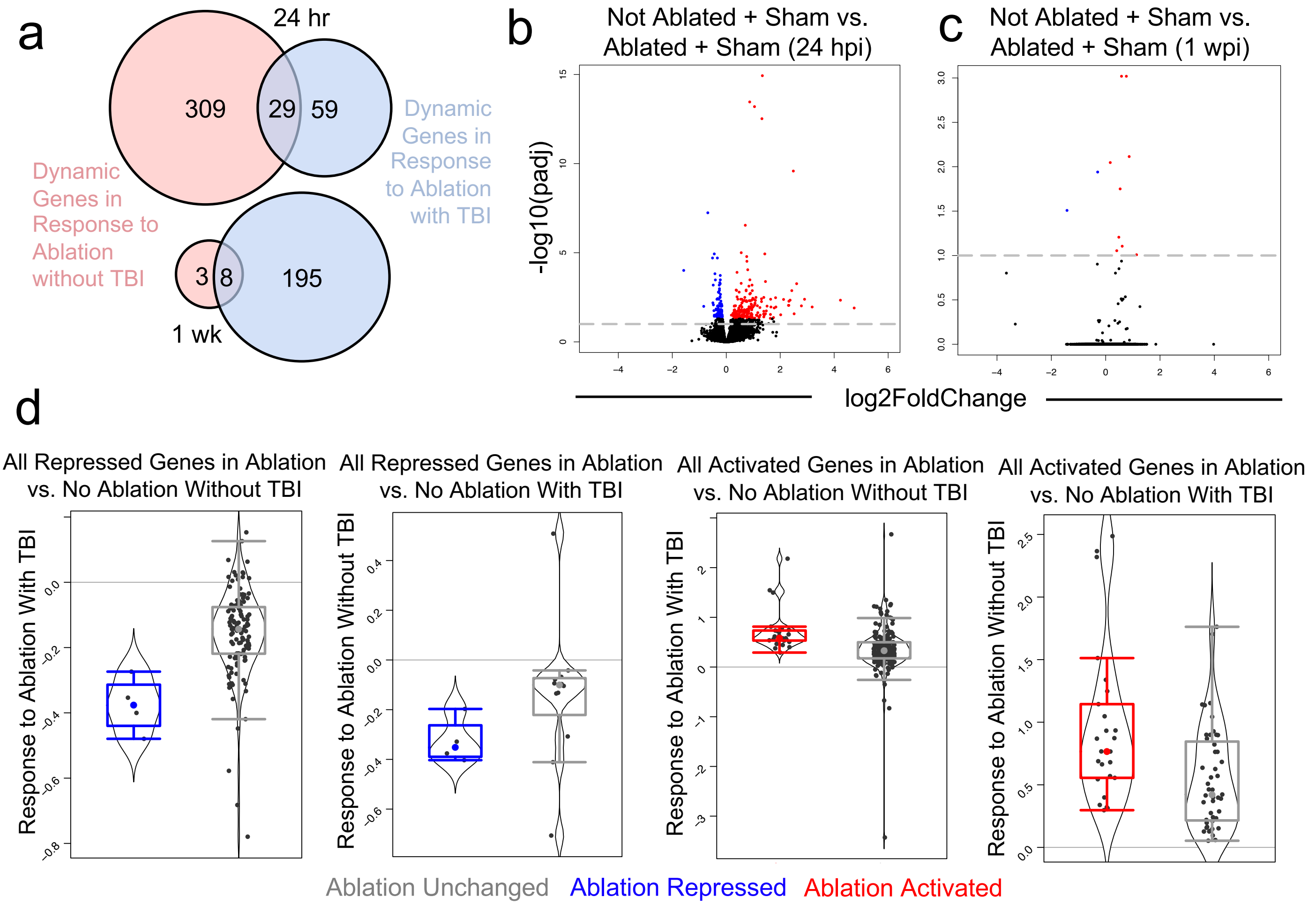

Supplementary Figure 6. Effects of photoablation on gene expression signatures. Mice were subjected to meningeal lymphatic photoablation after i.c.m. Visudyne injection or a control procedure. Mice were rested for one week and then underwent either TBI or a sham procedure. Bulk RNA sequencing was performed on these 4 experimental groups with 4 independent mice per group either 24 hours or 1 week post TBI. a) Total numbers of differentially expressed genes in response to Ablation without TBI (pink) in comparison to the total numbers of differentially expressed genes in response to Ablation with TBI (blue) at each sequencing time point (FDR<0.1). Shared genes are in overlapping sections. b-c) Volcano plots showing differentially expressed genes at (b) 24 hours post injury and (c) 1 week post injury in the Not Ablated + Sham vs. Ablated + Sham comparison (FDR <0.1). d) Box and whisker plots at 24 hours post injury depicting activated and repressed genes in response to ablation with and without TBI (FDR<0.1). The central dot within the box plots represents the median of the data set. The upper and lower boundaries of the box represent the third (Q3) and first (Q1) quartiles respectively. The length of the box therefore represents the interquartile range (IQR). The whiskers extend to the minimum and maximum values of the set, here defined as Q1 - 1.5*IQR and Q3 + 1.5 * IQR respectively. All data points beyond the whiskers are considered outliers. The box and whisker plots are overlaid on a violin plot that encompasses the entire data set, including the outliers. The width of the violin plot represents the frequency of observations at that given y-value. Therefore, the wider the violin plot, the higher the frequency of observations. FDR values were calculated with DEseq2 using the Wald test for significance following fitting to a negative binomial linear model and the Benjamini-Hochberg procedure to control for false discoveries.

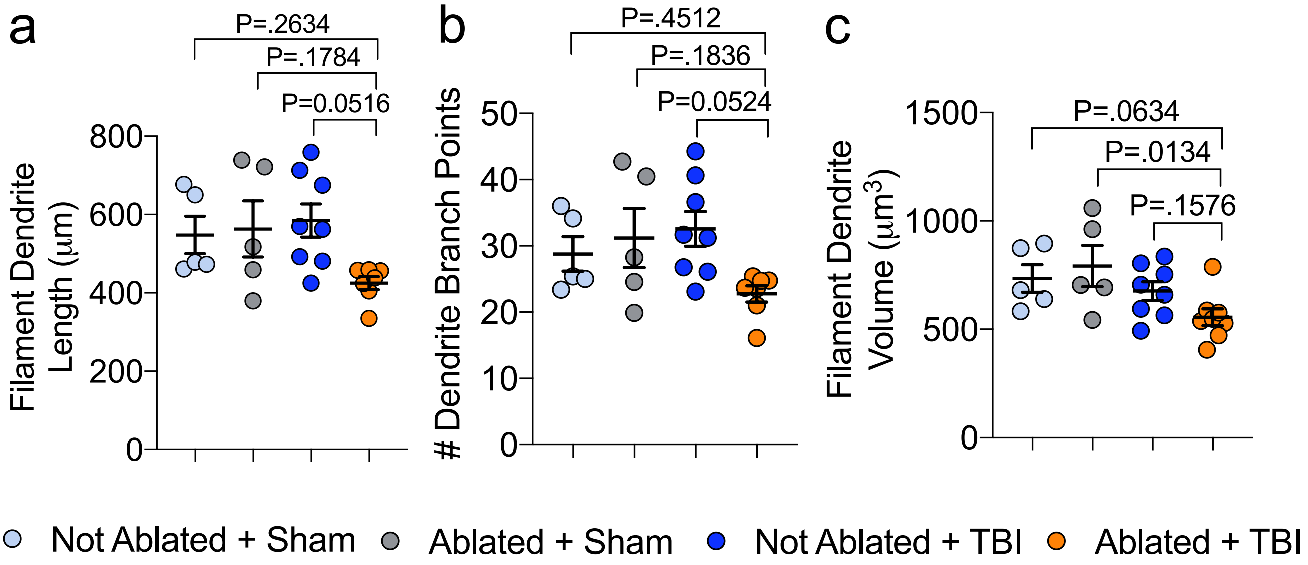

Supplementary Figure 7. The effect of pre-existing lymphatic dysfunction on Iba1+ cells. Mice were subjected to meningeal lymphatic photoablation after i.c.m. Visudyne injection or a control procedure and then to TBI or a sham procedure 1 week later. a-c) Brains were harvested 2 weeks after TBI, sectioned, and stained for Iba1. Quantification of the (a) average filament dendrite length (μm), (b) average number of dendrite branch points, and (c) average filament dendrite volume (μm^3) (Not Ablated+Sham n=5, Ablated+Sham n=5, Not Ablated+TBI n=8, Ablated+TBI n=7; data from one experiment). All n values refer to the number of mice used and the error bars depict mean ± s.e.m. *P* values calculated by two-way ANOVA with Tukey's multiple comparison correction (a-c). Source data (a,b,c) are provided as a Source data file.

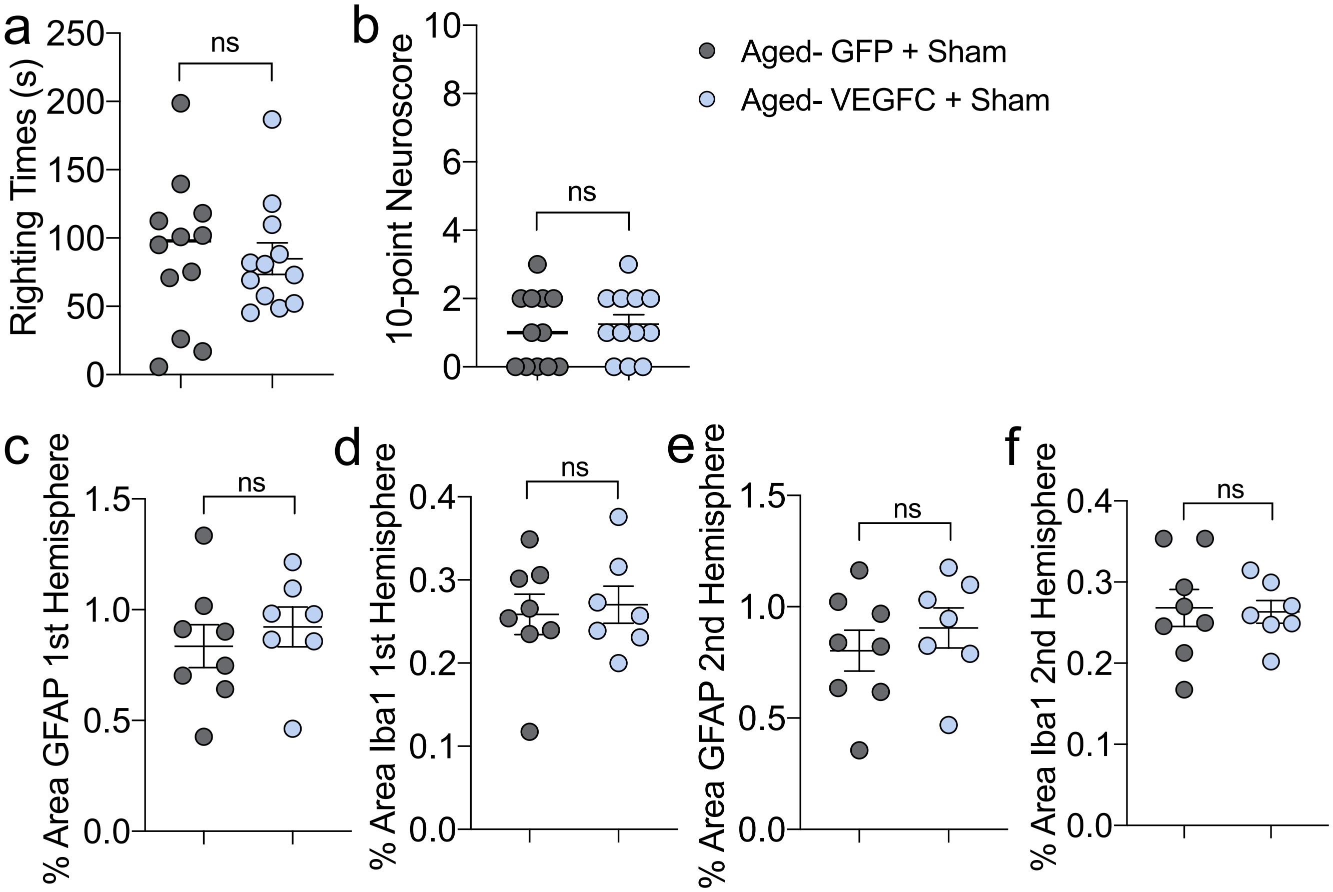

Supplementary Figure 8. Treatment of uninjured aged mice with VEGF-C does not influence righting time, neuroscore, or gliosis measures. Aged mice (18-20 months old) received 2 μl of artificial CSF containing 10^{13} genome copies per ml of either AAV1-CMV-mVEGF-C or control AAV1-CMV-eGFP by i.c.m. injection and then mice were subjected to sham treatment 2 weeks later. a) Righting times and b) 10-point gross neuroscore of mice at 1 hour post sham procedure (n=12 mice/group; pooled data from 2 independent experiments). Two weeks after sham procedure, brains were harvested, sectioned, and stained for markers of gliosis. c-f) Quantification of the percent area of Iba1 and GFAP immunoreactivity in both hemispheres of the brain (Aged-GFP+Sham n=8, Aged-VEGFC+Sham n=7). All n values refer to the number of mice used and the error bars depict mean ± s.e.m. *P* values were calculated using two-tailed Student's t-test. Source data (a,b,c,d,e,f) are provided as a Source data file.

Supplementary Table 1. Top 20 genes in the Not Ablated + TBI vs. Ablated + TBI group vs. Not Ablated + Sham vs. Ablated + Sham group

Gene	Not Ablated + TBI vs. Ablated + TBI (-log2padj)	Gene Response (Not Ablated + TBI vs. Ablated + TBI)	Not Ablated + Sham vs. Ablated + Sham (-log2padj)	Gene Response (Not Ablated + Sham vs. Ablated + Sham)	Fold Change of comparisons (Column 2/ Column 4)
X9630013A20Rik	26.970626	Activated	2.815854066	Activated	9.57813344
H19	21.1141876	Activated	22.06175671	Activated	0.95704925
Col1a1	15.4302398	Activated	30.38196062	Activated	0.50787505
Col1a2	10.7553359	Activated	30.98605305	Activated	0.34710248
C1qc	10.3289388	Activated	2.391067707	Activated	4.31980187
Mpeg1	6.75743072	Activated	1.756788511	Activated	3.84646796
C4b	5.98491719	Activated	4.616960161	Activated	1.29628955
Col6a1	5.98491719	Activated	3.999476138	Activated	1.49642528
Pld4	5.94028144	Activated	6.187779078	Activated	0.96000219
Tcirg1	5.45694594	Activated	4.717892655	Activated	1.15664903
Bgn	5.360354	Activated	10.40616731	Activated	0.51511319
Ctsh	5.14985031	Activated	3.157904469	Activated	1.63078091
Itgam	5.14985031	Activated	3.936637228	Activated	1.30818514
Gjc2	4.93885587	Activated	1.674230563	Activated	2.94992577
Ltbp4	4.93885587	Activated	1.657847813	Activated	2.97907675
Trem2	4.84161903	Activated	3.181598672	Activated	1.52175668
Col6a2	4.80695313	Activated	5.819750401	Activated	0.82597239
Fcrls	4.80695313	Activated	1.824303203	Activated	2.63495296
Irf7	4.80695313	Activated	2.971062138	Activated	1.61792413
Sema6a	4.80695313	Activated	0.991155235	Activated	4.8498489
Arc	13.0384876	Repressed	0.227624649	Repressed	57.2806491
Gng4	4.79734578	Repressed	0.494613486	Repressed	9.69918109
Hspa5	3.7987826	Repressed	4.253261616	Repressed	0.89314577
Gm43980	3.79364208	Repressed	0.058467469	Repressed	64.8846637
Pcsk1	3.79364208	Repressed	2.981308322	Repressed	1.2724756
Mras	3.6452025	Repressed	2.149743978	Repressed	1.69564494
Etv5	3.49060473	Repressed	10.8326543	Repressed	0.32222986
Zbtb7a	3.49060473	Repressed	3.151038462	Repressed	1.10776329
Rgs8	3.23818268	Repressed	0.402979251	Repressed	8.03560648
Serpinb8	3.1877807	Repressed	0.046380808	Repressed	68.7305981
Zfp324	3.16803996	Repressed	0.104637618	Repressed	30.2763005
Gfod1	3.15312218	Repressed	7.999403905	Repressed	0.39416964
Pdzd2	3.14330513	Repressed	0.660051173	Repressed	4.7622143
Spry4	3.06569035	Repressed	1.54188296	Repressed	1.98827695
Hyou1	3.05247886	Repressed	0.436498851	Repressed	6.99309713
Slc6a3	3.00642093	Repressed	0.14153804	Activated	21.2410807
Cbln4	2.94477885	Repressed	0.303388055	Repressed	9.70631112
Unc13c	2.87569266	Repressed	11.36028247	Repressed	0.25313567
Mex3c	2.81167812	Repressed	0.494613486	Repressed	5.68459655
Ppp1r10	2.59057765	Repressed	0.032459754	Activated	79.8089129
Mean	6.01042697	Repressed	4.830226859	Repressed	1.24433637
Median	4.80695313	Repressed	2.603460886	Repressed	1.70710307

Mice were subjected to meningeal lymphatic photoablation after i.c.m. Visudyne injection or a control procedure. Mice were rested for one week and then underwent either TBI or a sham procedure. Bulk RNA sequencing was performed on these 4 experimental groups with 4 independent mice per group either 24 hours or 1 week post TBI. Table depicts the negative log of the adjusted p-value of the top 20 up- and down-regulated genes from the Not Ablated + TBI vs. Ablated + TBI group as compared to the Not Ablated + Sham vs. Ablated + Sham group. The fold change of the comparisons shows the negative log of the adjusted p-value from the TBI comparison divided by the negative log of the adjusted p-value from the Sham comparison. FDR and P values were calculated with DEseq2 using the Wald test for significance following fitting to a negative binomial linear model and the Benjamini-Hochberg procedure to control for false discoveries. Source data are provided as a Source data file.
