# Supplementary material for: Meningeal lymphatic dysfunction exacerbates traumatic brain injury pathogenesis
Source: Nat Commun. 2020 Sep 10;11:4524. doi: 10.1038/s41467-020-18113-4 (PMC7483525; doi:10.1038/s41467-020-18113-4)
Supplement: Supplementary file 3 — Reporting Summary [file 41467_2020_18113_MOESM3_ESM.pdf]

nature research

Corresponding author(s): Lukens John, R

Last updated by author(s): Jul 27, 2020

Reporting Summary

Nature Research wishes to improve the reproducibility of the work that we publish. This form provides structure for consistency and transparency in reporting. For further information on Nature Research policies, see our Editorial Policies and the Editorial Policy Checklist.

Statistics

For all statistical analyses, confirm that the following items are present in the figure legend, table legend, main text, or Methods section.

n/a	Confirmed	
[ ]	[x]	The exact sample size (n) for each experimental group/condition, given as a discrete number and unit of measurement
[ ]	[x]	A statement on whether measurements were taken from distinct samples or whether the same sample was measured repeatedly
[ ]	[x]	The statistical test(s) used AND whether they are one- or two-sided<br>*Only common tests should be described solely by name; describe more complex techniques in the Methods section.*
[ ]	[x]	A description of all covariates tested
[ ]	[x]	A description of any assumptions or corrections, such as tests of normality and adjustment for multiple comparisons
[ ]	[x]	A full description of the statistical parameters including central tendency (e.g. means) or other basic estimates (e.g. regression coefficient) AND variation (e.g. standard deviation) or associated estimates of uncertainty (e.g. confidence intervals)
[ ]	[x]	For null hypothesis testing, the test statistic (e.g. F, t, r) with confidence intervals, effect sizes, degrees of freedom and P value noted<br>*Give P values as exact values whenever suitable.*
[x]	[ ]	For Bayesian analysis, information on the choice of priors and Markov chain Monte Carlo settings
[x]	[ ]	For hierarchical and complex designs, identification of the appropriate level for tests and full reporting of outcomes
[x]	[ ]	Estimates of effect sizes (e.g. Cohen's d, Pearson's r), indicating how they were calculated

Our web collection on statistics for biologists contains articles on many of the points above.

Software and code

Policy information about availability of computer code

Data collection	Noldus ethovision XT (version 14) was used to record the behavioral data in the Novel Location Recognition Test (NLRT). An accelerating rotarod (MED Associates Inc, ENV-575M) was used and the time spent on the rod and the speed at which the mouse fell or the trial ended was recorded using RotaRod Version 1.4.1, MED associates inc software. Intracranial pressure (ICP) was measured with a pressure sensor catheter (model SPR100, Millar). Imaging was conducted with a Lecia TCS SP8 confocal microscope and LAS AF software (version 3.5.5.19976, Leica Microsystems).
Data analysis	GraphPad Prism 8 was used to represent data in graphs and for the statistical analyses of the data. ImageJ/Fiji software (version 2.0.0-rc-69/1.52n) was used to outline and measure areas for imaging analysis. Microglia Sholl analysis was performed using Imaris software (9.5.1 Bitplane). For RNA sequencing data, the raw sequencing reads (FASTQ files) were aligned to the UCSC mm10 mouse genome build using the splice-aware read aligner HISAT2 (version 2.1.0). Samtools (version 1.10) was used for quality control filtering. Reads were sorted into feature counts with HTSeq (version 0.12.4). DESeq2 (version 1.26.0) was used to normalize the raw counts based on read depth and perform principal component analysis and differential expression analysis. The p-values were corrected with the Benjamini-Hochberg procedure to limit false positives arising from multiple testing. The gene set collections from MSigDB (version 7.1) were used for differential gene set enrichment analysis. The analysis itself was performed using the Seq2Pathway (version 1.18.0), fgsea (version 1.12.0), tidyverse (version 1.3.0), and dplyr (version 0.8.5) software packages. Heatmaps were generated using the pheatmap R package (version 1.0.12, https://github.com/raivokolde/pheatmap) while other plots were made with the lattice (version 0.20-41, http://lattice.r-forge.r-project.org/) or ggplot2 (version 3.3.0, https://ggplot2.tidyverse.org) packages. The GWAS Catalog was used to find genes associated with neurodegenerative or psychiatric diseases (https://www.ebi.ac.uk/gwas/home), and the circos plot including these data was generated using the circlize R package. Flow cytometry data was analyzed using FlowJo software (version 10.5.0, Treestar). Microsoft Excel (version 16.16.22) was used to compile data. All code used for analysis is available at [https://github.com/arun-b-dutta/TBI_Lymphatics_RNAseq-Analysis].

For manuscripts utilizing custom algorithms or software that are central to the research but not yet described in published literature, software must be made available to editors and reviewers. We strongly encourage code deposition in a community repository (e.g. GitHub). See the Nature Research guidelines for submitting code & software for further information.

Data

Policy information about availability of data

All manuscripts must include a data availability statement. This statement should provide the following information, where applicable:

- Accession codes, unique identifiers, or web links for publicly available datasets
- A list of figures that have associated raw data
- A description of any restrictions on data availability

The datasets generated and analyzed during the current study are available from the corresponding author on reasonable request. The source data underlying Figures 1-7 and Supplementary Figures 1-8 are provided as a Source Data file. Raw and processed sequencing data can be accessed through the Gene Expression Omnibus (GEO) at [https://www.ncbi.nlm.nih.gov/geo/query/acc.cgi?acc=GSE155063].

Field-specific reporting

Please select the one below that is the best fit for your research. If you are not sure, read the appropriate sections before making your selection.

[x] Life sciences [ ] Behavioural & social sciences [ ] Ecological, evolutionary & environmental sciences

For a reference copy of the document with all sections, see nature.com/documents/nr-reporting-summary-flat.pdf

Life sciences study design

All studies must disclose on these points even when the disclosure is negative.

Sample size	Sample sizes were chosen on the basis of standard power calculations (with α = 0.05 and power of 0.8).
Data exclusions	No data were excluded.
Replication	Key experiments were reiterated with representatives from each group with similar observations across iterations. Major experiments were repeated at least 2 times and trends were consistent across experiments.
Randomization	Within each iteration of an experiment, animals were randomly assigned to groups with approximately balanced sample size.
Blinding	Investigators were blinded as to experimental groups during data collection and analysis.

Reporting for specific materials, systems and methods

We require information from authors about some types of materials, experimental systems and methods used in many studies. Here, indicate whether each material, system or method listed is relevant to your study. If you are not sure if a list item applies to your research, read the appropriate section before selecting a response.

Materials & experimental systems

n/a	Involved in the study
[ ]	[x] Antibodies
[x]	[ ] Eukaryotic cell lines
[x]	[ ] Palaeontology and archaeology
[ ]	[x] Animals and other organisms
[x]	[ ] Human research participants
[x]	[ ] Clinical data
[x]	[ ] Dual use research of concern

Methods

n/a	Involved in the study
[x]	[ ] ChIP-seq
[ ]	[x] Flow cytometry
[x]	[ ] MRI-based neuroimaging

Antibodies

Antibodies used	anti-Lyve-1–eFluor 660 (eBioscience, ref:50-0443-82, clone: ALY7, 1:200, lot:2107832), anti-Lyve-1-eFluor 488 (eBioscience, ref:53-0443-82, clone: ALY7, 1:200, lot:2077936), anti-CD31 (Millipore Sigma, ref:MAB1398Z, clone: 2H8, 1:200, lot: 3099512), anti-Iba1 (Abcam, ref:ab5076, 1:300, lot:GR3253755-2), anti-GFAP (Thermo Fisher Scientific, ref:13-0300, clone: 2.2B10, 1:1000, lot:UE286801), donkey Alexa Fluor 594 anti-rat (Thermo Fisher Scientific, ref:A21209, 1:1000, lot:2078918), donkey Alexa Fluor 647 anti-goat (Thermo Fisher Scientific, ref:A21447, 1:1000, lot:2045332), and goat Alexa Fluor 488 anti-Armenian hamster (Jackson ImmunoResearch, ref:127-545-160, 1:1000, lot:136087).
Validation	All antibodies were validated by the manufacturer. anti-Lyve-1-eFluor 660: Reactivity, mouse. Usage, Flow, immunocytochemistry (ICC), immunofluorescence (IF), immunohistochemistry (IHC). anti-Lyve-1-eFluor 488: Reactivity, mouse. Usage, Flow, ICC, IF, IHC. anti-CD31: Reactivity, mouse, canine. Usage, Electron Microscopy (EM), flow, ICC, IF, IHC, Immunoprecipitation (IP), Neutralization Assay. anti-Iba1: Reactivity, mouse, rat, human. Usage, IHC, Western Blot (WB). anti-GFAP: Reactivity, guinea pig, human, mouse, rat,

bovine. Usage, WB, ELISA, IHC, ICC, IP, flow. donkey Alexa Fluor 594 anti-rat: Reactivity, rat. Usage, ICC, IF, IHC. donkey Alexa Fluor 647 anti-goat: Reactivity, goat. Usage, ICC, IF, IHC, WB. goat Alexa Fluor 488 anti-Armenian hamster: Reactivity, Armenian hamster. Usage, IHC.

Animals and other organisms

Policy information about studies involving animals; ARRIVE guidelines recommended for reporting animal research

Laboratory animals	Mice were housed and behavior was conducted in specific pathogen-free conditions under standard 12-hour light/dark cycle conditions in rooms equipped with control for temperature (21 ± 1.5°C) and humidity (50 ± 10%). Mice matched for sex and age were assigned to experimental groups and all adult mice used were between 8-10 weeks of age. Males and females were used for drainage and lymphangiogenesis studies, as sex has not been shown to influence lymphatic drainage at baseline [18]. Both males and females were also used to study the effects of increased ICP on lymphatic flow. Males were used for all pre-existing lymphatic dysfunction studies (Figures 4-6) for consistency with behavioral readouts and for consistency within the RNA sequencing data, as sex can influence both of these readouts. Male mice were also used for the aged mice experiments. C57BL/6J mice were obtained from Jackson Laboratories. All aged mice were between 18 and 24 months of age and were obtained from Jackson Laboratories and the National Institute on Aging (NIA) Aged Rodent Colonies.
Wild animals	Wild animals were not used in our studies.
Field-collected samples	Field-collected samples were not used in our studies.
Ethics oversight	All mouse experiments were performed in accordance with the relevant guidelines and regulations of the University of Virginia and approved by the University of Virginia Animal Care and Use Committee.

Note that full information on the approval of the study protocol must also be provided in the manuscript.

Flow Cytometry

Plots

Confirm that:

- [x] The axis labels state the marker and fluorochrome used (e.g. CD4-FITC).
- [x] The axis scales are clearly visible. Include numbers along axes only for bottom left plot of group (a 'group' is an analysis of identical markers).
- [x] All plots are contour plots with outliers or pseudocolor plots.
- [x] A numerical value for number of cells or percentage (with statistics) is provided.

Methodology

Sample preparation	Mice were euthanized and blood was collected by cardiac puncture. Red blood cells were lysed, and cells were then centrifuged for 5 minutes at 400 RCF and resuspended in 200 ul FACS buffer (pH 7.4; 0.1 M PBS; 1 mM EDTA, and 1% BSA).
Instrument	Gallios (Beckman Coulter)
Software	FlowJo software (Treestar)
Cell population abundance	The percent of microbeads present within all single cells in the blood were measured. The frequency ranged from 0.001 to 0.02.
Gating strategy	Single cells were gated using the height, area, and pulse-width of the forward and side scatter. Beads were gated based on their size and fluorescence.

- [x] Tick this box to confirm that a figure exemplifying the gating strategy is provided in the Supplementary Information.
